# Microvascular Structural Alterations in Cancer Patients Treated With Antiangiogenic Drugs

**DOI:** 10.3389/fcvm.2021.651594

**Published:** 2021-03-10

**Authors:** Maria Antonietta Coschignano, Carolina De Ciuceis, Claudia Agabiti-Rosei, Valeria Brami, Claudia Rossini, Giulia Chiarini, Paolo Malerba, Francesca Famà, Deborah Cosentini, Maria Lorenza Muiesan, Massimo Salvetti, Alina Petelca, Sara Capellini, Chiara Arnoldi, Matteo Nardin, Salvatore Grisanti, Damiano Rizzoni, Alfredo Berruti, Anna Paini

**Affiliations:** ^1^Department of Clinical and Experimental Sciences, University of Brescia, Brescia, Italy; ^2^Spedali Civili di Brescia, Clinica Medica University of Brescia and 2nd Division of Medicine, Brescia, Italy; ^3^Department of Medical and Surgical Specialties, Radiological Sciences, and Public Health, Medical Oncology, Spedali Civili di Brescia, University of Brescia, Brescia, Italy; ^4^Division of Medicine, Spedali Civili di Brescia, Brescia, Italy

**Keywords:** VEGF, hypertension, microcirculation, small resistance arteries, capillaroscopy, retinal arterioles, adaptive optics, oncology

## Abstract

**Objective:** Antiangiogenic therapies (tyrosine kinase inhibitors-TKI and direct anti-VEGF monoclonal antibodies) are being increasingly used in the treatment of solid tumors; hypertension represents a common side effect of these agents. Several mechanisms are involved in the development of hypertension, including microvascular rarefaction and other microvascular alterations. Therefore, the aim of our study was to evaluate whether TKI and direct anti-VEGF agents may affect the structure of retinal arterioles or capillary density.

**Design and Methods:** We investigated 20 patients with a diagnosis of cancer who underwent a treatment with either a TKI or an anti-VEGF antibody. Patients were submitted to ambulatory monitoring blood pressure for blood pressure evaluation. Basal and total capillary density were assessed by capillaroscopy whereas, retinal arteriole morphology was measured by Adaptive Optics. Patients were evaluated before starting the antiangiogenic therapy (T0) and re-evaluated after 3 (T3) and 6 (T6) months after treatment. Fourteen patients completed the study.

**Results:** Systolic and diastolic blood pressure values were similar in all patients at T3 and T6 compared to T0. However, during the study antihypertensive treatment was optimized (increased dose and/or addition of drugs) in 57% of patients (*n* = 8). No differences were observed in retinal arteriole structural parameters and in large artery stiffness. Basal capillary density was reduced by antiangiogenic drugs after 3 or 6 months.

**Conclusions:** Our data suggest that an increase of antihypertensive treatment is necessary in patients treated with a TKI or a direct VEGF inhibitor, confirming pro-hypertensive effects of these drugs. However, under adequate blood pressure control, microvascular structure seem to be partially preserved, since a worsening of basal capillary density but no changes in retinal arteriole morphology were observed.

## Introduction

Angiogenesis (the formation of new blood vessels from a pre-existing vascular system) represents an essential mechanism for tumor growth and dissemination (1). Already in 1971 it was hypothesized the existence of a factor responsible for tumor angiogenesis that could be a target for the development of new antineoplastic therapies (2). In the last decades, our understanding of the molecular mechanism involved in angiogenesis grew steadily, and this led to the development of new treatments for solid tumors, namely drugs that targeted the pro-angiogenic factor vascular endothelial grow factor (VEGF). The inhibition of angiogenesis has significantly improved the prognosis for several solid tumors (3).

### Inhibition of Angiogenesis and Hypertension

Vascular homeostasis is regulated by various pro-angiogenesis and anti-angiogenesis factors. When the pro-angiogenetic factors pre-dominate angiogenesis begins; and when this occurs in a tumoral context this is called “angiogenic switch” (4). As mentioned, one of the most important pro-angiogenic factors is VEGF; there are different isoforms of this factor, but the most biologically relevant is VEGF-A (5). The effect of VEGF is mediated by interaction with VEGF receptors (VEGFR) (6). The interaction between VEGF-A and its receptor produces various effects and, in particular, stimulates the proliferation, differentiation and migration of endothelial cells as well as the production of nitric oxide. Different drug classes that inhibit the action of VEGF-A are presently available. Bevacizumab, a humanized monoclonal antibody that inhibits circulating VEGF-A and prevents its interaction with the receptor was approved in 2004 for the treatment of colon cancer (7).

Ramucirumab is another monoclonal antibody which acts at the level of the extracellular portion of the VEGF receptor. Multitarget small molecules tyrosine kinase inhibitors (TKI) may block multiple tyrosine kinases, including VEGFRs 1, 2, and 3; stem cell factor receptor (c-KIT); platelet-derived growth factor receptors (PDGFRs) and other tyrosine kinases (8).

Treatment with these drugs is frequently associated with an increase in blood pressure both in hypertensive patients and in normotensive subjects (9, 10). Hypertension is dose-dependent and varies in incidence among the different angiogenesis-inhibitor drugs (10). The mechanism by which VEGF-inhibitors may elevate blood pressure in human remain uncertain; however, it is hypothesized that an impairment of nitric oxide signal pathway with endothelial dysfunction and increased oxidative stress, an activation of endothelin system and microvascular rarefaction may be involved (10). All these events would favor an increase in peripheral resistance.

Several evidences suggested that an increase in blood pressure, in patients treated with VEGF inhibitors, could be considered a pharmacodynamic biomarker of oncologic response (11, 12), although this was not confirmed by other studies (13). However, poorly controlled hypertension is associated with cardiovascular events, and it may also lead to discontinuation of anticancer therapy, thus, potentially limiting the overall clinical benefit.

### Microcirculation and Hypertension

It is known that essential hypertension is associated with the presence of structural alterations in the microvessels (14, 15). Resistance arteries exposed to high pressure values undergo over time a remodeling process characterized by an increase in the vascular wall thickness together with a reduction in the internal diameter (14–16). The result of these alterations is the increase in the ratio between the media thickness and the lumen of the vessel (MLR: media/lumen ratio) (14–16). This index is currently considered the main indicator of the extent of hypertensive remodeling in the microcirculation, and it was shown to be an important predictor of cardiovascular events (16). MLR is usually evaluated by micromyographic approaches which are considered as the gold standard technique for assessing the microvascular alterations in humans (17). In particular, this technique was used for the evaluation of the morphology and function of small arteries obtained from biopsies of the subcutaneous fat tissue. However, this approach is limited by the local invasiveness of the procedure. In recent years, non-invasive evaluation of the retinal microcirculation as a mirror of the cerebral and coronary vascular system has aroused increasing interest (18). A novel and promising technological approach in this regard approach became recently available, that is to say the direct measurement of wall to lumen ratio (WLR) of retinal arterioles using an adaptive optics imaging system (18). With this technique it is possible to obtain very high quality images of the retinal arterioles that allow a direct measure of the WLR in precise and reproducible way (18).

Besides structural alterations of small resistance arteries, in hypertensive patients there are also other microvascular changes, including: endothelial dysfunction, increased oxidative stress, and capillary rarefaction (16). In the capillary district the absolute number of perfused vessels contributes to total vascular resistance. Vascular rarefaction is defined either as a functional rarefaction (when vessels are temporarily not perfused) or anatomical rarefaction (when vessels are permanently absent) (16, 17). Capillary rarefaction can be assessed non-invasively by videomicroscopy/capillaroscopy which allows evaluating the basal and total capillary density in various regions of the skin (nailfold, dorsum of fourth finger's non-dominant hand and forearm) (16, 17). Total capillary density is obtained through venous congestion (16, 18).

Finally, it was suggested that treatment with VEGF inhibitors may increase large artery stiffness, increasing pulse wave velocity (PWV) (19), although the presence of an altered vascular distensibility was not confirmed in other studies (20).

Therefore, the aim of our study was to evaluate whether TKI and direct anti-VEGF agents may have an impact on microvascular and macrovascular structure, in particular on retinal arteriolar morphology and on capillary density.

## Patients and Methods

Twenty patients, 10 males and 10 females, with diagnosis of cancer were included in the study. Demographic and clinical characteristics were detailed in Table 1.

**Table 1:** Demographic and clinical characteristics of the patients enrolled.

	***N* = 20**
Age (years)	66 ± 9.5
Gender (males/female)	10/10
Body mass index (Kg/m^2^)	23.2 ± 3.6
Smoking status: active/previous/no smoking **(n,%)**	2/7/11, 10%/35%/55%
Previous history of hypertension *(n,%)*	9, 45%
Previous history of diabetes mellitus *(n,%)*	2, 10%
Previous history of dyslipidaemia *(n,%)*	3, 15%
Previous history of ischemic heart disease *(n,%)*	1, 5%
Familiarity for cancer *(n,%)*	10, 50%
Familiarity for cardiovascular disease *(n,%)*	10, 50%
Kidney tumor *(n,%)*	9, 45%
Lung tumor *(n,%)*	3, 15%
Gastrointestinal tumor *(n,%)*	6, 30%
Thyroid tumor *(n,%)*	1, 5%
Breast cancer *(n,%)*	1, 5%
Treatment with sunitinib *(n,%)*	4, 20%
Treatment with pazopanib *(n,%)*	6, 30%
Treatment with bevacizumab *(n,%)*	4, 20%
Treatment with nintedanib *(n,%)*	2, 10%
Treatment with ramucirumab *(n,%)*	2, 10%
Treatment with lenvatinib *(n,%)*	1, 5%

Patients were enrolled between January 2017 and December 2019 in the Oncology Department of our hospital. All patients were evaluated before starting antitumoral treatment (T0) after 3 months (T3) and after 6 months (T6) of treatment.

One patient, after an initial evaluation at T0 was then excluded from the study because he was no longer suitable, from an oncological point of view, for the previously proposed therapy. Five patients were lost during the follow-up between T0 and T3 (*n* = 1 due to change in cancer therapy and *n* = 4 due to worsening of clinical conditions secondary to neoplastic pathology). Therefore, the data analysis was carried out on the remaining 14 patients.

Routine blood chemistry tests were performed, according to standard clinical oncological follow-up. At T0, T3, and T6, patients underwent a 24-h non-invasive blood pressure monitoring, a capillary density assessment by capillaroscopy, and a retinal circulation assessment by adaptive optics.

### 24-h Ambulatory Blood Pressure Monitoring and Evaluation of Large Artery Distensibility

Twenty-four hour ambulatory blood pressure monitoring ABPM was performed in all patients using Mobil-O-Graph® (IEM GmbH, Aachen, Germany) for evaluation of 24-h day-time and night-time average pressure values, according to Italian (21) and European (22) guidelines. The Mobil-O-Graph is an oscillometric device, whose brachial blood pressure-detection unit was validated according to standard protocols (23, 24). Among various indexes, the device calculates augmentation pressure and augmentation pressure (AP), as well as central systolic blood pressure (cSBP), central diastolic blood pressure (cDBP), central pulse pressure (cPP), and PWV. Briefly, after recording brachial BP, cuff re-inflates at diastolic phase for ~10 s and records brachial pulse waves with a high-fidelity pressure sensor (25). Brachial BP is used for calibration of the pulse waveform. Then, the software reconstructs the aortic pulse waveform by means of the ARCSolver algorithm using a generalized transfer function, as previously described (26, 27). Wave separation analysis is also performed by decomposing the aortic pulse waveform into forward-traveling (incident) and backward-traveling (reflected) pulse waves with a triangular aortic flow waveform (25).

The device calculates: cSBP and cDBP, estimated as the levels of SBP and DBP at the aorta, based on the aortic pulse wave generated by the generalized transfer; augmentation pressure (AP), estimated as the difference of the pressure at second minus the pressure at first inflection point of the systolic phase of pulse wave; AP, and heart rate-adjusted AP [AP(75)], indicative of the augmentation component of aortic SBP because of the premature arrival of the reflected wave; PWV, estimated from the reconstructed aortic pulse waveform via mathematical models taking into account the characteristic impedance and age and assuming a three-element Windkessel model (25–27). Previous validation studies in hypertensive and healthy volunteers showed acceptable agreement between Mobil-O-Graph-derived parameters and invasive measurements or non-invasive readings using applanation tonometry, with a slight underestimation of PWV with the Mobil-O-Graph device (28, 29).

### Capillaroscopy/Videomicroscopy

Capillary density, defined as the number of capillaries for unit of skin area, was evaluated trough a capillaroscopy (Videocap 3, DS Medica, Milan, Italy) before and after venous congestion. The examination was performed under standardized conditions: before the start of the procedure the patient was kept at rest in a sitting position in a quiet room at a controlled temperature (21–22°C). The capillaries of the nailfold were analyzed (first row of nail bed capillaries) and the dorsum of the fourth finger of the non-dominant hand using a fiber optic video microscope, in basal conditions (basal capillary density) and after venous congestion (total capillary density) in order to visualize the capillaries functionally excluded. Venous congestion was achieved by inflating a cuff of a pediatric sphygmomanometer at 60 mmHg applied to the base of the fourth finger of the non-dominant hand. In addition, the basal and total capillary density at the level of the distal third of the forearm was also evaluated using a standard sphygmomanometer to obtain venous congestion. Further details are reported in reference (30).

### Evaluation of the Retinal Microcirculation

All enrolled patients underwent study of retinal arteries with adaptive optics using the Rtx-1 optical camera (Imagine Eyes, Orsay, France) (18, 30). The camera is able to recognize and correct the aberrations of a light beam entering the eye through a closed system consisting of a superluminescent diode and an adaptive optics system. At the time of acquisition, the camera obtains 40 images in 4 s, returning images of the retinal fundus with correction of the aberrations produced by the dioptric means, and therefore at a much higher resolution than any other camera for the study of the fundus oculi (18, 30). The measurements were taken in a sitting position after 5 min of rest. The superior temporal portion of the optic disc of the right retina was examined. With a specific software the internal and external diameter of the arterioles were measured and then wall cross sectional area (WCSA) and WLR ratio were then calculated (18, 30). The measurement of WLR using adaptive optics was previously demonstrated to be closely correlated with the measurement of MLR of subcutaneous small arteries (30) that, as mentioned, is considered the gold standard methods of assessment of microvascular alterations and is a potent predictor of prognosis (16, 17).

In all patients an echocardiographic assessment and an ultrasonographic investigation of the carotid arteries was performed at baseline. An echocardiogram was repeated in 10 patients during the follow up period. Left ventricular mass index, systolic and diastolic function, as well as the possible presence of intima/media thickening of the carotid artery were evaluated, according to previously published methods and criteria (31).

To quantify carotid artery wall thickness, the following measures were chosen: mean of the maximum wall thickness of 2 sites (far wall of the left and right side) for common carotid; the maximum of 2 sites (far wall of the left and right side) for common carotid; mean of the maximum of 4 sites (far wall of the left and right side) for common carotid and bifurcation (CB Max); mean of the maximum of all 12 sites (4 sites at each of the three segments), or of at least 4 sites for each side: CA Mean Max); the maximum of all 12 sites (4 sites at each of the three segments), or of at least 4 sites for each side.

The protocol of the study was approved by the Ethics committee of our Institution, and informed consent was obtained from each participant. The procedures followed were in accordance with institutional guidelines.

### Statistical Analysis

All data are expressed as mean ± standard deviation. All parameters showed a normal distribution. Quantitative variables were evaluated by Student's *t*-test comparing T0 with T3 and with T6; a one-way ANOVA analysis of variance (ANOVA) (comparison between three time points) was also performed. Statistical significance between groups was assessed by means of chi-square test for dichotomous variables. A value <0.05 was considered as statistical significant. By a *post-hoc* analysis, our study had a 75% power to detect a difference of 0.02 in the WLR, while it has a power >98% to detected a difference of at least 0.03 in the WLR, than on the bases of previous data may be considered as clinically relevant (16).

## Results

Demographic and clinical characteristics are summarized in Table 1, while laboratory parameters and need for treatment for arterial hypertension in the patients enrolled are reported in Table 2.

**Table 2:** Laboratory parameters and antihypertensive treatment in the patients enrolled.

	**T0 (Baseline)**	**T3 (3 months)**	**T6 (6 months)**
Serum creatinine (mg/dl)	0.98 ± 0.4	0.92 ± 0.35	0.97 ± 0.41
Serum glucose (mg/dl)	95.6 ± 11.9	97.3 ± 10	95.1 ± 13.6
Total serum cholesterol (mg/dl)	196.7 ± 43.7	162.7 ± 32.7	183.2 ± 18.3
Serum triglycerides (mg/dl)	135.6 ± 41.8	161.5 ± 91.3	119.2 ± 43.1
Treatment for arterial hypertension	8/20, 40%	11/14, 78%	12/13, 92%^**^

** *Chi-square test: p < 0.01*.

### ABPM

**Figure 1:**
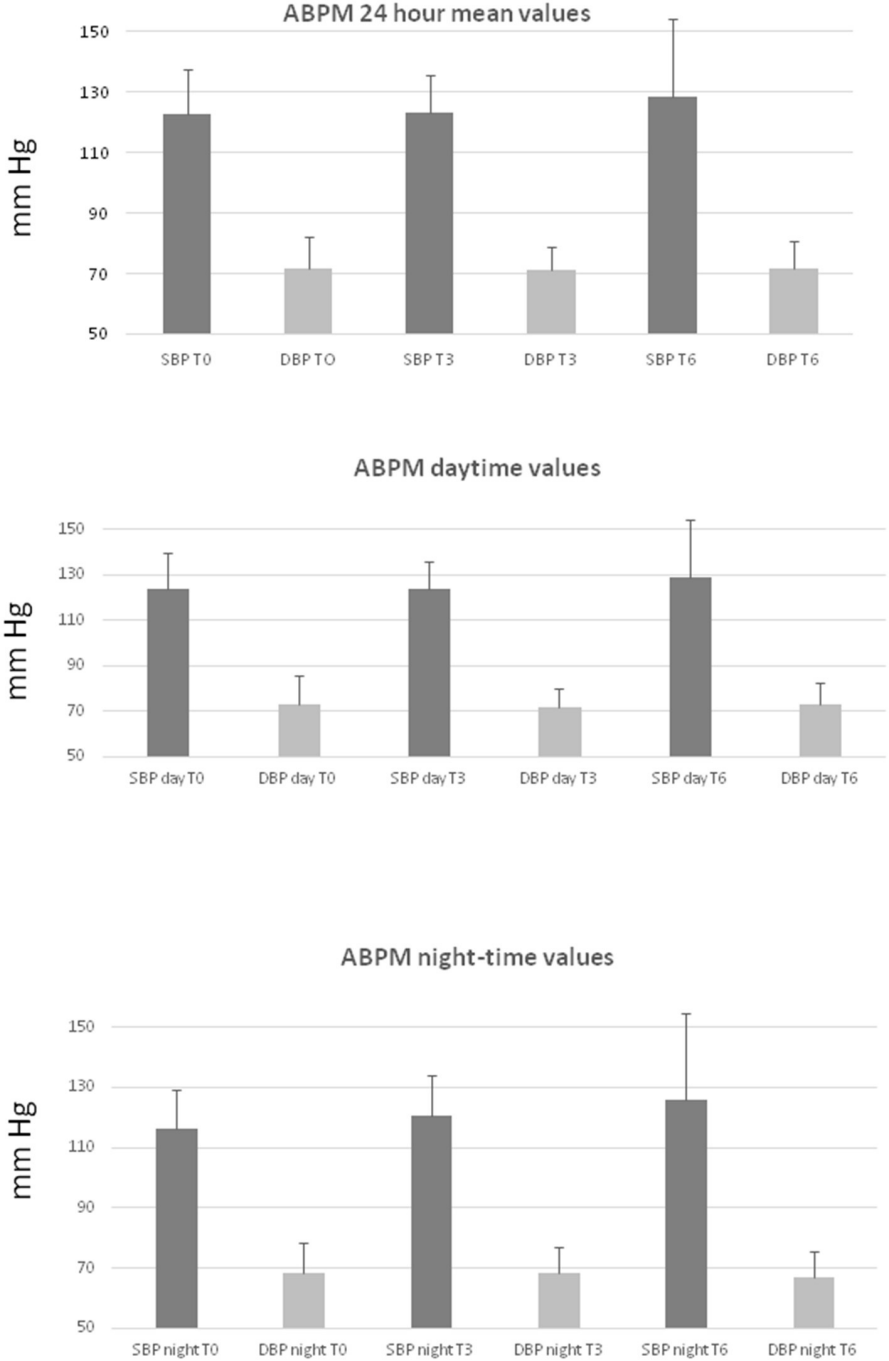
Blood pressure values from ambulatory blood pressure monitoring (ABPM). Top panel: systolic (SBP) and diastolic (DBP) mean 24 h values; middle panel: systolic (SBP day) and diastolic (DBP day) daytime values; bottom panel: systolic (SBP night) and diastolic (DBP night) night-time values at the different time points (T0, T3, T6). P = NS between time points. Data are expressed as mean + standard deviation.

Results are summarized in Table 3, Figure 1. No statistically significant difference in systolic and diastolic blood pressure values were observed at T3 or T6 compared with T0 (Table 3, Figure 1). However, during the study it was necessary to optimize the antihypertensive treatment with dosage increased or the introduction of new treatments in 57% of patients (*n* = 8 out of 14). In particular, in the first 3 months from the beginning of the antiangiogenic therapy it was necessary to increase the dosage of the drugs for the control of blood pressure values in four patients, while in three patients an antihypertensive therapy was introduced *ex novo* and only one patient required a reduction in antihypertensive therapy. Between the third and sixth month, two patients further increased the antihypertensive therapy and in one patient antihypertensive drug treatment was started. The therapeutic changes occurred when an elevation of blood pressure (<140/90 mm Hg) was detected by the general practitioner, regardless of the blood pressure assessments performed during the study. The drugs used were, mainly, dihydropyridine calcium antagonists, and angiotensin converting enzyme (ACE) inhibitors.

**Table 3:** Blood pressure values and indices of microvascular structural alterations at the different time points.

**Parameter**	**T0 (Baseline)**	**T3 (3 months)**	**T6 (6 months)**
**ABPM**			
24-h systolic blood pressure (mmHg)	122.6 ± 14.7	123.3 ± 12.2	128.3 ± 26.1
24-h diastolic blood pressure (mmHg)	71.7 ± 10.2	70.9 ± 8.0	71.7 ± 9.1
**Retinal arterioles**			
Internal diameter (μm)	86.4 ±17.0	87.14 ±11.4	89.3 ±11.8
External diameter (μm)	111.5 ±21.6	111.9 ± 15.0	114.4 ± 14.7
Wall thickness (μm)	12.6 ±2.6	12.4 ± 2.0	12.6 ± 1.8
Wall cross sectional area (μm^2)^	4,039.6 ± 1,491	3,943.5 ± 1,093.0	3,968.3 ± 1,026
Wall to lumen ratio (WLR)	0.30 ± 0.03	0.28 ± 0.02	0.28 ± 0.03
**Capillaries**			
Basal capillary density (dorsum), number per area unit	72.0 ± 15.8	65.5 ±18.6^*^	61.9 ± 17.1^#^
Total capillary density (dorsum), number per area unit	73.8 ± 14	69.6 ± 20.7	68.5 ± 11.9
Basal capillary density (forearm), number per area unit	56.6± 17.1	47.7 ± 13.2^§^	49.3 ± 11.6
Total capillary density (forearm), number per area unit	58.4 ± 16.2	54.5 ± 15.8	53.0 ± 8.0

*T-student analysis*:

* *T3 vs. T0 p = 0.03*;

# *T6 vs. T0 p = 0.02*;

§ *T3 vs. T0 p = 0.04*.

*One-way ANOVA: dorsum basal capillary density p = 0.027, forearm basal capillary density p = 0.17*.

### Retinal Arterioles

Results are summarized in Table 3. There were no differences in the parameters evaluated for the assessment of microvascular alterations in the retina, and in particular in the WLR at T3 and T6 compared to baseline (Table 3).

### Capillary Density

**Figure 2:**
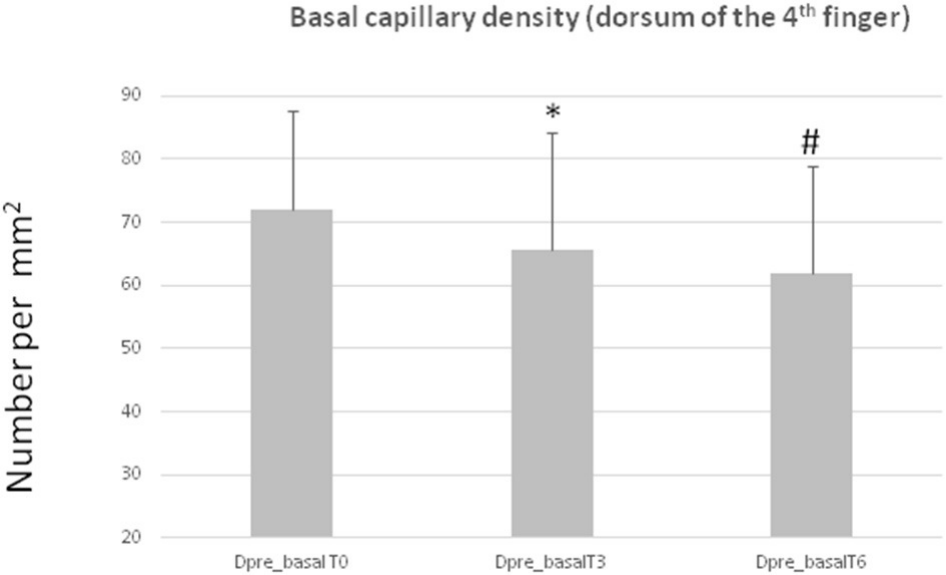
Basal capillary density in the dorsum of the 4th finger (Dpre_basal) at the different time points (T0, T3, T6). *T3 vs. T0 *p* = 0.03; #T6 vs. T0 *p* = 0.02. Data are expressed as mean + standard deviation.

Results are summarized in Table 3. A significant reduction in the basal capillary density in the dorsum of the 4th finger was observed after 3 and 6 months of antiangiogenic treatment compared to baseline (Table 3, Figure 2). This was also confirmed by the one-way ANOVA (Table 3). A significant reduction was also seen in the basal capillary density of the forearm after 3 months of therapy. There were no significant reductions in the total capillary density in any site as well as in the basal capillary density in the nailfold.

### Macrocirculation

No significant difference was observed between visits in terms of indices or large artery distensibility. Central blood pressures, (cSBP, cDBP, PWV, AP and AP75) were similar at the different time points (Table 4).

**Table 4:** Large artery distensibility and cardiovascular parameters at the different time points.

**Parameter**	**T0 (Baseline)**	**T3 (3 months)**	**T6 (6 months)**
PWV (m/sec)	13.1 ± 3.10	13.6 ± 3.08	14.4 ± 4.11
AP (mm Hg)	26.0 ± 13.5	29.8 ± 12.8	26.1 ± 15.9
AP(75) (mm Hg)	21.6 ± 9.42	25.8 ± 9.70	24.5 ±8.8
Central SBP (mm Hg)	129 ± 28.6	126 ± 13.8	132 ± 25.4
Central DBP (mm Hg)	81.7 ±13.8	80.1 ± 6.00	86 ± 11.7
Central PP (mm Hg)	47.5 ± 17.2	45.6 ± 9.64	46.5 ± 18.2
Left ventricular hypertrophy *(n, %)*	1/13, 7.7%	1/10, 10%	2/11, 18%
LVMI (g/m^2^)	80.0 ± 19.0	85.3 ± 14.1	82.8 ± 18.2
LVMh^2.7^ (g/m)	36.0 ± 9.30	38.8 ± 7.98	36.6 ± 9.86
Relative wall thickness	0.37 ± 0.06	0.38 ± 0.02	0.37 ± 0.05
Ejection fraction (%)	60.6 ± 4.62	61.1 ± 5.22	69.7 ± 5.20
Cardiac output (L/min)	3.84 ± 1.32	3.63 ± 1.57	3.54 ± 1.22
Cardiac index ((L/min/m^2^)	1.06 ± 0.34	0.93 ± 0.28	0.93 ± 0.20
e velocity (cm/sec)	68.5 ± 19.2	65.5 ± 21.6	58.7 ± 21.5
a velocity (cm/sec)	91.5 ± 23.8	83.2± 31.1	72.4 ± 24.7
e/a	0.75 ± 0.11	0.88 ± 0.35	0.86 ± 0.28
E-wave deceleration time (msec)	222 ± 46.9	257 ± 40.1^*^	240 ± 51.7^#^
IVRT (mm/sec)	88.1 ± 9.9	96.6 ± 30.7	97.6 ± 13.0^§^
Carotid artery intima/media thickening *(n, %)*	12/13, 92%	NA	NA
CA MeanMax (mm)	1.31 ± 0.30	NA	NA
CB Max (mm)	1.36 ± 0.33	NA	NA

*AP, augmentation pressure; SBP, systolic blood pressure; DBP, diastolic blood pressure; PP, pulse pressure; LVMI, left ventricular mass index; LVMh, ventricular mass indexed for height; IVRT, isovolumic deceleration time; CA MeanMax, mean maximum intima-media thickness of internal, external carotid artery and bifurcation; CB Max, maximum intima-media thickness of external carotid artery and bifurcation; NA, not available*.

*T-student analysis*:

* *T3 vs. T0 p = 0.04*;

# *T6 vs. T0 p = 0.03*;

§ *T6 vs. T0 p = 0.01*.

### Cardiovascular Parameters

The majority patients (92%) had intima-media thickening of the carotid artery at baseline (intima media thickness > 1mm) (31). No change in left ventricular mass were detected at the different time points (Table 4), while diastolic function (E-wave deceleration time, isovolumic deceleration time) tended to worsen after 3 and 6 months of antiangiogenetic therapy (Table 4). No change in indices of systolic function was observed (Table 4).

## Discussion

Our results showed that, during a 6-month follow-up in neoplastic patients treated with VEGF inhibitor drugs or TKI, no statistically significant alterations were observed for the WLR of retinal arterioles; there was a not statistically significant increase in blood pressure values, evaluated by ABPM as well. However, these results were deeply influenced by changes in antihypertensive treatment (starting a *de novo* treatment, increase in the number or in the dose of previous antihypertensive treatment independently from the study evaluations. In fact, eight patients developed an increase in blood pressure values during the follow up period (three were on treatment with pazopanib, one with lenvatinib and four with sunitinib). Probably such prompt therapeutic adjustment allowed both to effectively control blood pressure, with no significant increases at the two time points and also to prevent the development of microvascular damage in the retinal vessels.

The reduction in capillary density at the dorsum of the finger and in the forearm may be ascribed to the antiangiogenic effect of the drugs, which seemed to be independent of antihypertensive therapy.

A reduction of capillary density was previously observed in some (32, 33), but not in other studies (20). In our study we did not observe any change in total capillary density during treatment, and no change was observed for basal or total capillary density in the nailfold. The nailfold capillary district was demonstrated to be less informative in terms of changes in the presence of cardiovascular risk factors (34, 35). In addition, the number of patients evaluated in our study is relatively small, and this could have led to a loss of statistical power. This may also has been the reason why we could observe a reduction in basal capillary density (i.e., spontaneously open capillaries), but not in total capillary density (i.e., spontaneously + forced open capillaries). An alternative explanation would be that functional, rather than anatomical alterations are involved.

Antiangiogenetic drugs therefore promoted the development of arterial hypertension or in any case a worsening of blood pressure control in patients who were already hypertensive, but a correct monitoring of the blood pressure and therefore a prompt start or titration of antihypertensive treatment, may avoid the development of microvascular damage at least in terms of increase of the WLR ratio at the retinal level. This may be clinically important, since microvascular damage, in particular an increased MLR in the subcutaneous small resistance arteries (and probably also an increased WLR in the retinal arterioles) is associated to organ damage (36) and it is known to be an important predictor of cardiovascular events, associated with a reduced event-free survival (16). A significant correlation between coronary flow reserve and subcutaneous small resistance artery remodeling has been detected in hypertensive patients, suggesting that structural alterations may be present at same time in small resistance arteries and in different other vascular beds including the brain (16, 36–38). There is a general consensus about the deleterious effect of antiangiogenetic agents on vascular structure, due to vasotoxic effects of these drugs (39). As mentioned, in our study we evaluated possible microvascular alterations in the retinal district with a very reliable and precise method (18, 30, 40).

In our study, no change in mechanical properties of large arteries (distensibility, stiffness) was observed during treatment with VEGF inhibitor drugs, similar to what observed by Dalbeni et al. (20). Similarly, no changes in cardiac structure and function were observed, except for a slight worsening of left ventricular relaxation. Also in this case, careful blood pressure control during treatment may have been of help in avoiding the development of cardiac and macrovascular alterations.

Our neoplastic patients were mainly treated with ACE inhibitors and calcium channel blockers, according to indication of European Guidelines (41). Consensus documents or guidelines on the management on this clinical condition are in any case, still scarce (10, 42).

As mentioned, a limitation of this study is certainly related to the small number of the patients enrolled, possibly exposing to a type 2 error. They had often advanced neoplastic conditions and therefore not all patients were able to complete even a relatively short 6-month follow-up. Only 9 of the 20 enrolled patients were alive 1 year after T0 (11 patients died for the worsening of the neoplastic disease). In addition, in the study protocol no blood chemistry test was scheduled; therefore the reported values were collected retrospectively: for this reason we were not able to perform a correct stratification of the patients' initial cardiovascular risk, or to accurately assess the trend of biohumoral indices over time.

Antiangiogenic drugs are widely used in cancer patients, however to date no predictive parameters of efficacy have been identified. Since the efficacy of these drugs could be linked to the extent of the antiangiogenic effect, the assessment of capillary density could be a non-invasive and easily assessable parameter that could be of help in detecting patients destined to respond to treatments and those who do not. This could be an interesting topic for future research.

The prognostic impact of changes in blood pressure and/or in vascular morphology is not known in these patients, also considering that life expectancy is very variable on the bases of baseline prognostic factors. The patients evaluated in our study are representative enough of the usual patients undergoing such treatments. However, at least some of them may survive longer enough, and in these patients the impact of hypertension/vascular alterations may became a clinical issue.

In conclusion, our data confirm the importance of paying attention to the cardiovascular risk of the patients in antiangiogenic therapy, in particular by maintaining periodic blood pressure monitoring. The need for increased antihypertensive therapy during treatment with TKI or anti-VEGF is confirmed, confirming the pro-hypertensive effects of these drugs. However, in the presence of adequate blood pressure control, no changes in the retinal microcirculation are observed, thus underlining the importance of controlling cardiovascular risk factors also in these patients both for the prevention of events and to ensure greater compliance to the antineoplastic therapy itself.

## Data Availability Statement

The raw data supporting the conclusions of this article will be made available by the authors, without undue reservation.

## Ethics Statement

The studies involving human participants were reviewed and approved by Ethics Committees, Spedali Civili di Brescia. The patients/participants provided their written informed consent to participate in this study.

## Author Contributions

MC: clinical handling of patients, data collection, and graphics. CD: design, data analysis, and graphics. CA-R, VB, CR, GC, PM, FF, APe, SC, CA, MN, and APa: data collection. DC and SG: clinical handling of patients. MM, MS, and AB: design and data analysis. DR: data analysis and writing of the manuscript. All authors contributed to the article and approved the submitted version.

## Conflict of Interest

The authors declare that the research was conducted in the absence of any commercial or financial relationships that could be construed as a potential conflict of interest.
